# Experimental Study on Force Sensitivity of the Conductivity of Carbon Nanotubes-Modified Epoxy Resins

**DOI:** 10.3390/ma11071174

**Published:** 2018-07-10

**Authors:** Chun-Feng Wan, Bo Wen, Jian-Guo Dai, Jian-Xun Liu, Yu-Zhong Liu

**Affiliations:** 1 Key Laboratory of Concrete and Prestressed Concrete Structure of Ministry of Education, Southeast University, Nanjing 210096, China; bowennj@hotmail.com (B.W.); llljjjxxxun@126.com (J.-X.L.); liuchongppp@163.com (Y.-Z.L.); 2 Department of Civil and Environmental Engineering, The Hong Kong Polytechnic University, Hong Kong 999077, China; cejgdai@polyu.edu.hk; 3 Jiangsu Advanced Institute of Seismic Resistant Technology for Mechanical and Electrical Equipment, Xingzhuang Industrial Park, Lishui District, Nanjing 211200, China

**Keywords:** carbon nanotubes, epoxy resin, force sensitivity, mixing amount, resistivity

## Abstract

The addition of a conductive material into polymer improves its mechanical properties, electrical properties and thermal conductivity and bestows it with good self-sensing and self-adjusting properties. In this study, carbon nanotubes-modified epoxy resins (CNTs-EP) were successfully prepared with good dispersion through the combined methods of three roller rolling, ultrasonic processing and adding surfactant. Tests were conducted to evaluate the resistivity of unloaded modified epoxy resins with different mixing amounts of carbon nanotubes (CNTs), to determine the conductive percolation threshold. On the basis of the test results, a series of monotonic and cyclic uniaxial tensile tests were then conducted to investigate the force sensitivity of the conductivity of epoxy resins modified with different mixing amounts of CNTs. The relationship between the stress and the resistivity under various mixing amounts was studied, indicating that the resistance response could play a good warning role on the damage of the modified polymer material.

## 1. Introduction

The application scope of polymer matrix materials has expanded from the aerospace and military industries to commercial airplanes, automobiles, civil structures and leisure/sport equipment by using them to replace traditional ceramic and metal-based materials [1,2,3]. The addition of a conductive material into polymer can improve its mechanical properties, electrical properties and thermal conductivity and entitles it with good self-sensing and self-adjusting properties [4]. The modified polymer material will be refactored under external forces within the conductive pathway, indicating certain force sensitive properties. Due to their good flexibility and processability, polymer conductive composites are often used as a sensing material in force sensitive sensors. The modified polymer materials own the broad application prospects and important research values in the field of structural health monitoring [5].

The discovery of nanomaterials has opened a new prelude for the study of nanosized sensing materials. Among which, the carbon nanotubes (CNTs) own a larger aspect ratio, a larger specific surface and better conductivity even with a lower mixing amount [6,7], under which CNTs show better conductivity while enhancing the mechanical properties of the polymer [8,9]. The modified polymer materials composed of CNTs present excellent sensitivity to force [10,11]. Since the size of CNTs is in nano-scale, there is an obvious tunnel effect between particles, which can be used to improve the sensitivity. Therefore, there exist broad research prospects on the use of CNTs in force sensitive composites [12]. Producing conductive polymer nanocomposites with a small amount of CNTs dispersed in insulating polymers then becomes possible and such electrically conductive CNT/polymer nanocomposite can be applied to various fields, including piezoresistive and high sensitive resistance-type strain sensors [13,14,15].

The dispersion of CNTs in epoxy resin (EP) was studied by researchers and a variety of physical and chemical methods of CNTs dispersion have been put forward [16,17,18,19,20]. Regarding the electrical properties of CNTs-EP, a lot of studies have been done to achieve a lower conductive percolation threshold in the matrix and verified that a low mixing amount of CNTs can make the epoxy resin more conductive [21,22,23,24]. Mechanical tests have also been conducted on the resin matrices modified with different mixing amounts of CNTs. It was found that a certain amount of CNTs can effectively enhance the mechanical properties of the matrix material such as the elastic modulus and the tensile strength [25,26]. However, research on the force sensitivity of CNTs-EP is still limited.

In this study, a series of experimental studies were conducted on CNTs-EP to investigate the sensitivity of their conductivity on force. Uniaxial tensile tests and cyclic loading tests were performed on the specimens with various mixing amounts of CNTs to reveal the relationship between the mixing amount and the force sensitivity of the conductivity of the modified epoxy resins.

## 2. Materials and Methods

### 2.1. Material Selection and Preparation Process

The materials required in specimen preparation mainly included epoxy resin, CNTs, dispersant and short carbon fiber powder. The descriptions of the materials are summarized in Table 1.

Carbon nanotube is a kind of nanoscale material with a large ratio of length to diameter and high surface energy. In the process of its dispersion into the resin composite matrix, aggregation phenomenon usually occurs, which will create difficulty for the formation of conductive network and then greatly affect the conductivity. Therefore, to find an optimal dispersion method for CNTs is the key to the successful preparation of CNTs-EP.

The dispersion methods of CNTs can be divided into two categories: physical and chemical dispersion methods. The physical dispersion methods mainly include high-energy ball grinding, ultrasonic processing, three roller rolling and centrifugal dispersion. The chemical dispersion methods mainly contain adding surfactant and strong acid and alkali washing. In this study, the used resin matrix belonged to a type of structural adhesive with high viscosity. It is therefore difficult to achieve good dispersion with the simple grinding or ultrasonic processing method. On the other hand, the introduction of other solvents such as acetone may affect the mechanical properties of structural adhesive and the added solvents are difficult to be completely removed afterward. In this experimental study, a dispersant activator was firstly added into the CNTs and the epoxy resin (i.e., part A) and the mixture was grinded with a three-roll machine. Secondly, a curing agent (i.e., part B) and short carbon fiber powder were added. The uniformly stirred short carbon fiber with the length of 1 mm and the diameter of 7 um was used to optimize the conductivity of the modified matrix.

**Figure 1:**
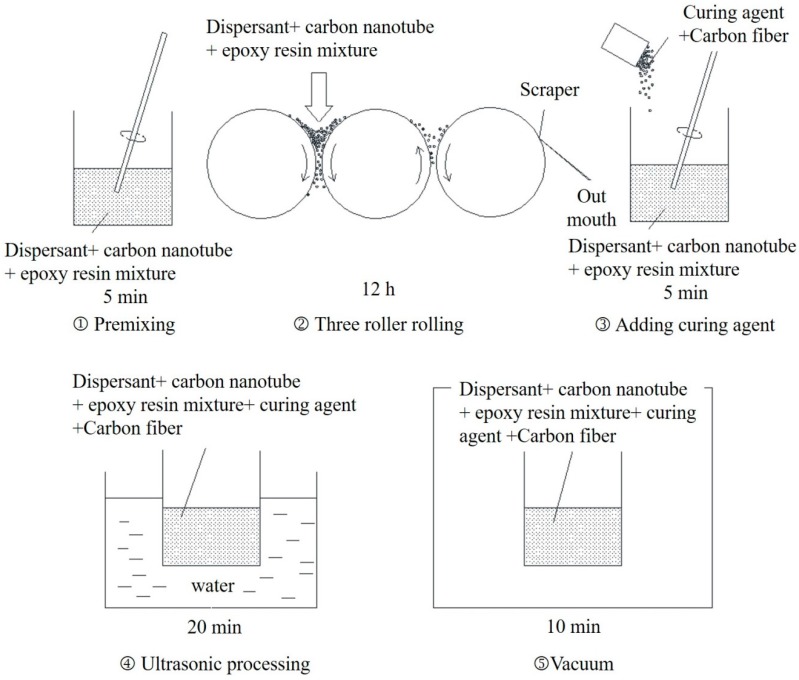
Preparation process of modified epoxy resin.

The mixture was further dealt with the normal temperature ultrasonic processing for the full dispersion of CNTs. Finally, the method of vacuum was used to remove air bubbles. The above preparation process is schematically illustrated in Figure 1.

### 2.2. Specimen Fabrication

The shape of the tensile specimens of the carbon nanotube-modified epoxy resin was dumbbell with a total length of 220 mm. In the central part of 60 mm length, the cross section was a rectangular with the dimension of 10 mm × 4 mm, while at the two ends of 53 mm length, the cross section was also rectangular but with the dimensions of 20 mm × 4 mm. For the transition part in between the length was 26.9 mm and the chamfer radius were 75 mm.

**Figure 2:**
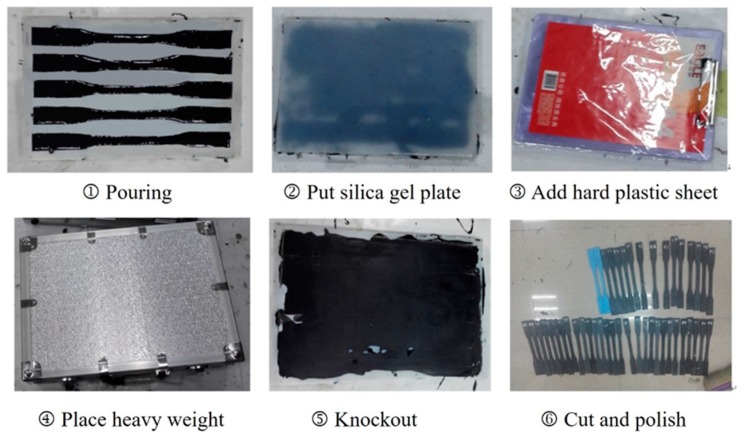
Preparation process of epoxy resin coupons.

Firstly, the modified epoxy resin was inserted into silica gel mold and a silica gel plate was used to seal the surface of the mold. Then, a hard plastic sheet was added to the silica gel plate and heavy weight was then placed on the sheet to ensure a close connection between the silica gel plate and the mold. At the room temperature of 20 + 0.5 °C, the mixtures were cured for 7 days. Then the specimens were cut and polished. All the specimens were measured by a Vernier caliper. Figure 2 shows the detailed fabricating process of the specimens.

### 2.3. Test Method

The conductivity of CNTs-EP is expected to be improved. The jointed CNTs in the matrix may form a conductive network and adjacent particles or aggregation particles of CNTs can form channel current or electric current under the excitation of an electric field. These two electronical transfer ways improve the conductive property of the modified epoxy resin matrix.

**Figure 3:**
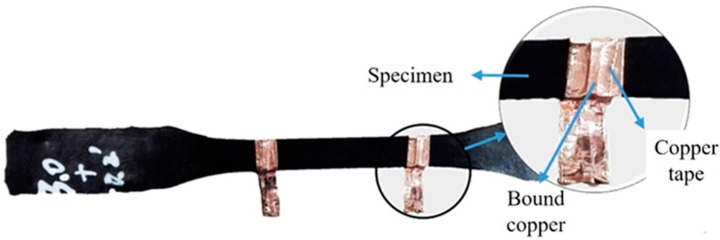
The electrodes attached to the epoxy coupon.

In the resistivity test of the modified epoxy resin matrix, two-point measurement was adopted. A copper sheet of 2 mm width was wound around the surface of the specimen and then a double-sided conductive copper tape of 6 mm width was attached onto the copper sheet. In this way, two electrodes were made and attached to the central part of the specimen with an interval of 50 mm. Figure 3 presents the locations of the two electrodes along the test coupon.

**Figure 4:**
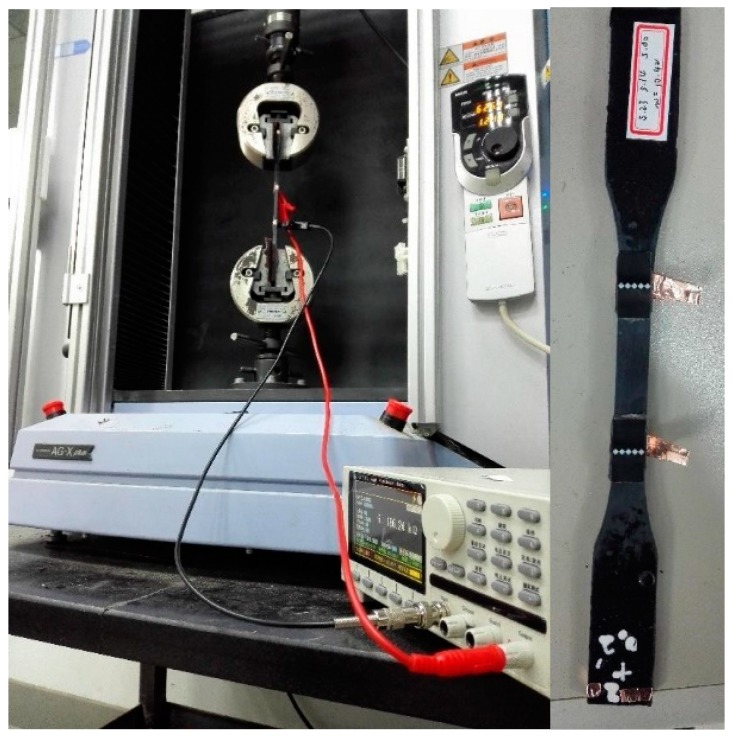
Test setup.

The test equipment was an electro-mechanical universal testing machine AG-Xplus (Shimadzu Co. Ltd., Kyoto, Japan). The electric resistance measuring instrument was an insulation resistance tester CHT3530 (Hopetech Co. Ltd., Shenzhen, China). In this study, monotonic and cyclic uniaxial tensile tests were conducted, respectively. The loading rate was fixed at 2 mm/min. In the monotonic uniaxial tests, the elastic modulus, elongation and tensile strength could be obtained. As for the cyclic loading tests, the maximum loading was set approximately at 2/3 of the tensile failure load obtained in the monotonic uniaxial tensile test. During the test process, the time-dependent stress, strain and electrical resistance were all automatically recorded. Considering the possible interference of the metal clip of the universal testing machine on the electrical performance of the specimens, the gripping part of the specimens was wrapped by a coarse sand paper (the resistance of the sand paper is in the magnitude of TΩ), to prevent the electrical connection of the specimen ends with the metal clip. The test set up is shown in Figure 4.

## 3. Results and Discussion

### 3.1. Resistivity of Modified Epoxy Resin

**Figure 5:**
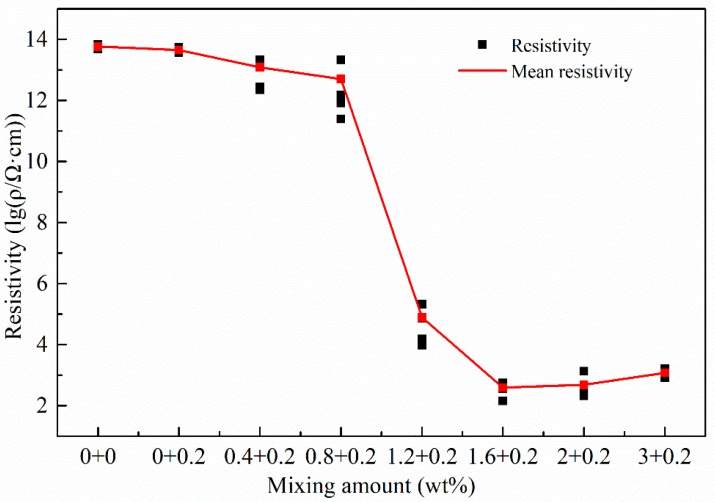
Resistivity test results of modified specimens with various mixing amounts of carbon nanotubes (CNTs) (with the constant mixing amount of 0.2 wt % for carbon fiber powder).

The resistivity tests were divided into seven groups with different mixing amounts of CNTs 0.0 wt %, 0.4 wt %, 0.8 wt %, 1.2 wt %, 1.6 wt %, 2.0 wt % and 3.0 wt %. On the other hand, the mixing amount of carbon fiber powder was fixed at 0.2 wt % for all the specimens. There were 5 identical specimens tested for each combination of test parameters, leading to 35 specimens in total. Figure 5 shows the resistivity test results.

The test results showed that without CNTs, the addition of carbon fiber powders had little influence on the resistivity of the specimens compared to the pure resin case. However, when the mixing amount of CNTs increased from 0.0 wt % to 0.8 wt %, the resistivity of the specimens dropped slowly. The conductive particles were still isolated during this stage and the resistivity of the composites was measured at the magnitude of TΩ·cm. When the mixing amount of CNTs increased further from 0.8 wt % to 1.2 wt %, the resistivity fell sharply by about 8 magnitudes. In this range, the conductive particles adjoined and the space between particles decreased, improving the tunnel effect among the particles and shifting the modified resin matrix from an insulator to a conductor. So, 1.2 wt % might be the conductive percolation threshold of the composite material. With the increase of mixing amount from 1.2 wt % to 1.6 wt %, the decrease of the resistivity became less sharp while in the range of 1.6 wt % to 2.0 wt %, the change of resistivity kept marginal, indicating that the conductive network in the modified matrix was sufficiently connected. When the mixing amount exceeded 2.0 wt %, a slightly increase of the resistivity was even seen probably due to the aggregation phenomenon of the CNTs. In other words, a very high mixing amount may lead to worse dispersion of the CNTs and thus weaken the electrical conductivity of the modified matrix.

**Figure 6:**
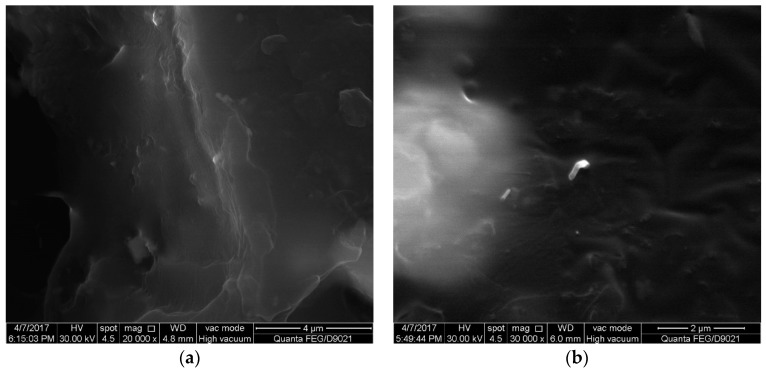

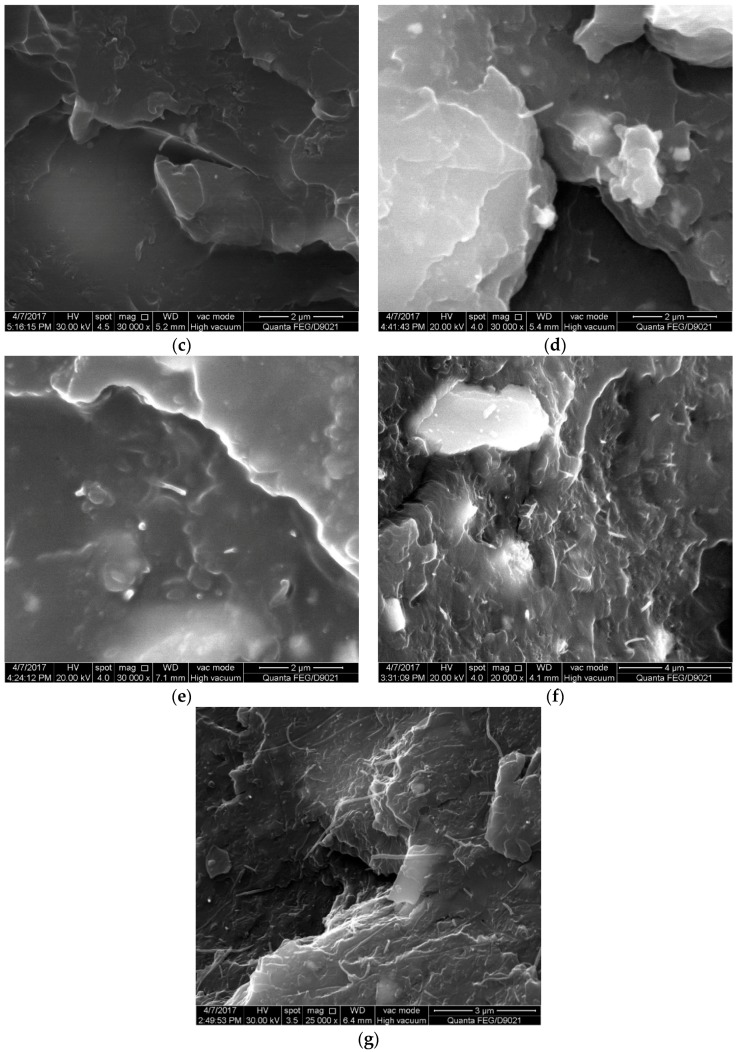
Scanning electron microscope (SEM) images of the modified matrix with various mixing amounts of CNTs: (**a**) 0.0 wt %; (**b**) 0.4 wt %; (**c**) 0.8 wt %; (**d**) 1.2 wt %; (**e**) 1.6 wt %; (**f**) 2.0 wt %; (**g**) 3.0 wt %.

For further explanation of the test results, the scanning electron microscopy (SEM) was performed to investigate the morphological characterization as shown in the Figure 6a–g. With the increase of the mixing amount of CNTs, it is seen that the white particles and filaments gradually increase which represents the added CNTs. At the mixing amount of 2.0 wt % the CNTs dispersed uniformly in the epoxy resin matrix in the form of network, which presents a good dispersion property. In Figure 6f, the aggregation phenomenon occurred at the mixing amount of 3.0 wt %. With the increase of the mixing amount of CNTs, the viscosity of the resin matrix increased and the fluidity decreased, which are consistent with many other previous investigations [27,28].

**Figure 7:**
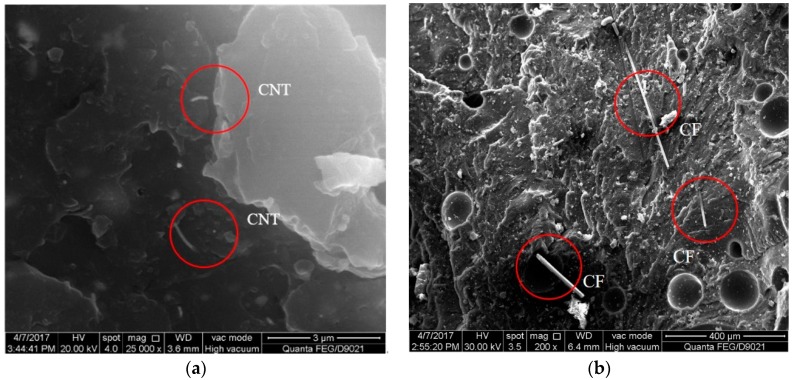
SEM images of the specimen with the mixing amount of 0.8 wt %: (**a**) CNTs; (**b**) carbon fibers.

**Figure 8:**
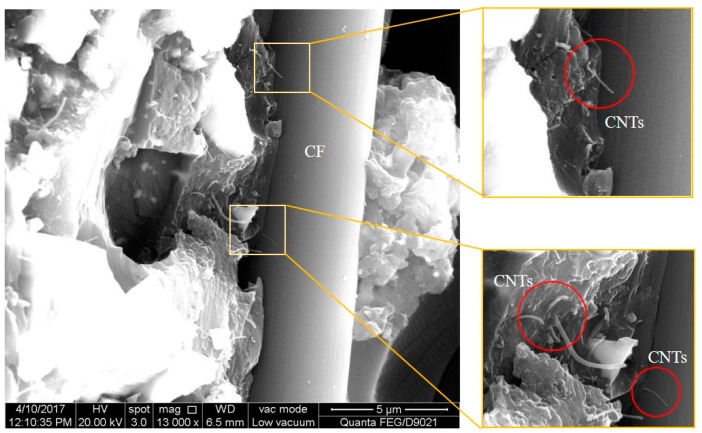
SEM images of the specimen with the mixing amount of 1.2 wt %.

With the mixing amount of 0.8 wt % and 1.2 wt %, the specimens were polished and then SEM was conducted for further morphological investigation. The corresponding results are shown in Figure 7 and Figure 8. As illustrated in Figure 7, at the mixing amount of 0.8 wt %, the specimen surface appeared to have many short carbon fibers. The carbon fibers dispersed homogeneously in the modified matrix in conjunction with the CNTs. This benefited the formation of the conductive network. There were also junction regions between carbon fibers and CNTs at the mixing amount of 1.2 wt %, as shown in Figure 8.

### 3.2. Force Sensitivity in Monotonic Uniaxial Tensile Tests

**Figure 9:**
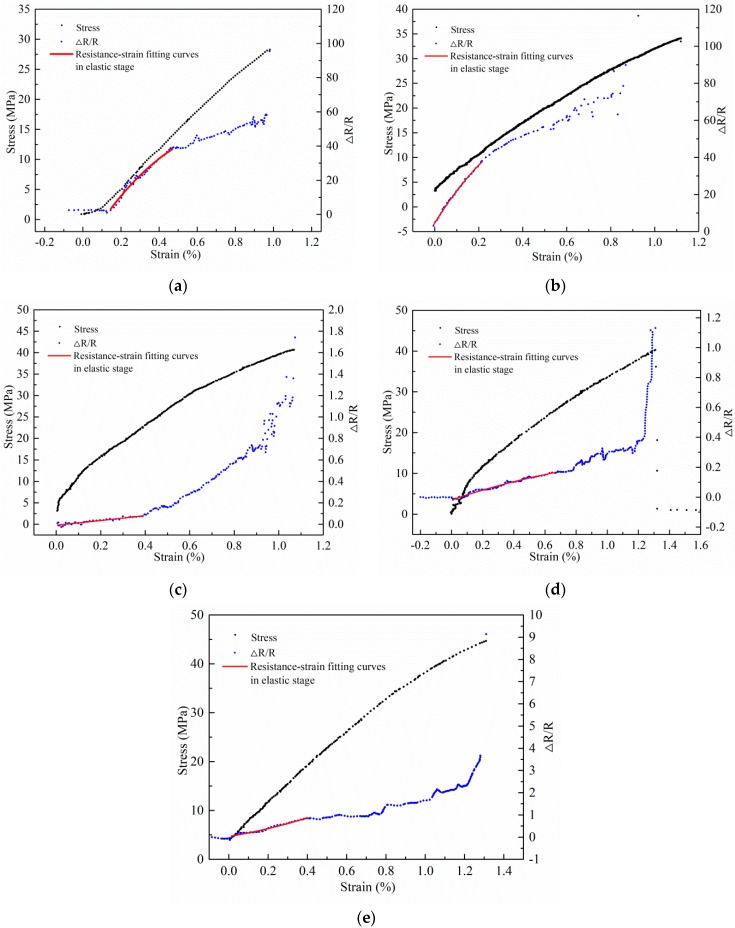
Results of monotonic uniaxial tensile tests: (**a**) 0.4 wt %; (**b**) 0.8 wt %; (**c**) 1.2 wt %; (**d**) 1.6 wt %; (**e**) 2.0 wt %.

Figure 9 shows the tensile stress-strain curves and the relative resistance-strain curves of specimens with various mixing amounts of CNTs (0.4 wt %, 0.8 wt %, 1.2 wt %, 1.6 wt %, 2.0 wt %), which were obtained from the monotonic uniaxial tensile tests. Here the relative resistance ΔR/R is denoted as the ratio of the resistance change to the initial resistance. It should be noted in Figure 9 that some piezoresistive instability existed at the initial stages of the curves. Such instability was removed during the regression analysis of the curves.

The improvement of conductivity of modified epoxy resin matrix by addition of CNTs was mainly composed of two mechanisms: (1) the Ohmic contacted the conductive network formed by the CNTs in the matrix, which can be explained by the seepage theory [29]. The segment resistance *R_c_* of the conductive network can be used to characterize such effect; (2) the conductive path formed by the other adjacent CNTs, which can be explained by the tunnel effect theory. The tunneling junction resistance *R_j_* between adjacent CNTs can be used to characterize such effect.

As displayed in Figure 9, for the mixing amounts of 0.4 wt % and 0.8 wt %, the relative resistance-strain curves are in a convex shape and the gauge factors are high (Figure 9a,b). Here, the slope of the resistance-strain curves in the elastic stage is used to define the gauge factors. The gauge factors under various mixing amounts are summarized in Table 2. When the tensile strain is small, the modified matrix is in the elastic stage. Because of the small mixing amount of CNTs, the jointed CNTs were less and the conductive network was not formed. The conductivity of the modified matrix mainly depended on the conductive path formed by tunnel effect of the other adjacent CNTs. The junction resistance *R_j_* between adjacent CNTs played a leading role. At the micro level, the spacing of CNTs particles had a great influence on the junction resistance *R_j_*. Therefore, at the macro level, the resistance was very sensitive to the strain change. The relative resistance value increased obviously with the increase of strain. The gauge factors are in high values. When the strain continued to increase, there was a certain level of damage occurring in the modified matrix. At the micro level, the spacing of CNTs particles exceeded the tunneling distance. As a result, at the macro level, the relative resistance became less sensitive to the strain change and the increase of resistance was slowed down.

When the mixing amounts were 1.2 wt %, 1.6 wt % and 2.0 wt %, the relative resistance-strain curves are in a concave shape. The gauge factors reduce and the linearity increases. Under the small tensile strain, the modified matrix was in elastic stage. Due to the larger mixing amount of CNTs, the connected internal conductive network was perfect and contributed significantly to the conductivity of the modified matrix. The segment resistance *R_c_* played a leading role. The number of jointed CNT particles decreased linearly with the increase of the strain, so there was a linear increase of the relative resistance value with the increase of the strain and the gauge factors are relatively in low values. With the continuous increase of the stain, microcracks occurred in the modified matrix, inducing and result in the fluctuation of the relative resistance values.

### 3.3. Force Sensitivity in Cyclic Uniaxial Tensile Tests

**Figure 10:**
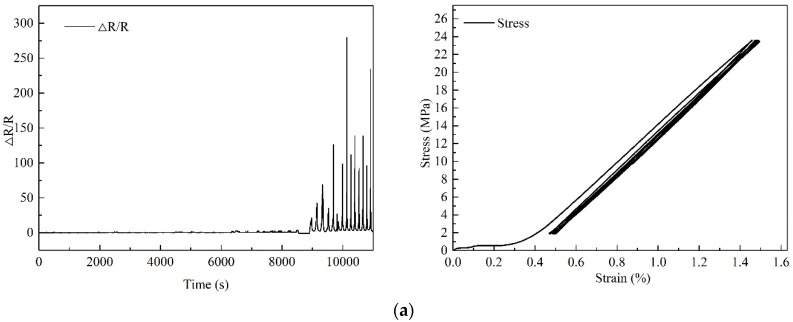

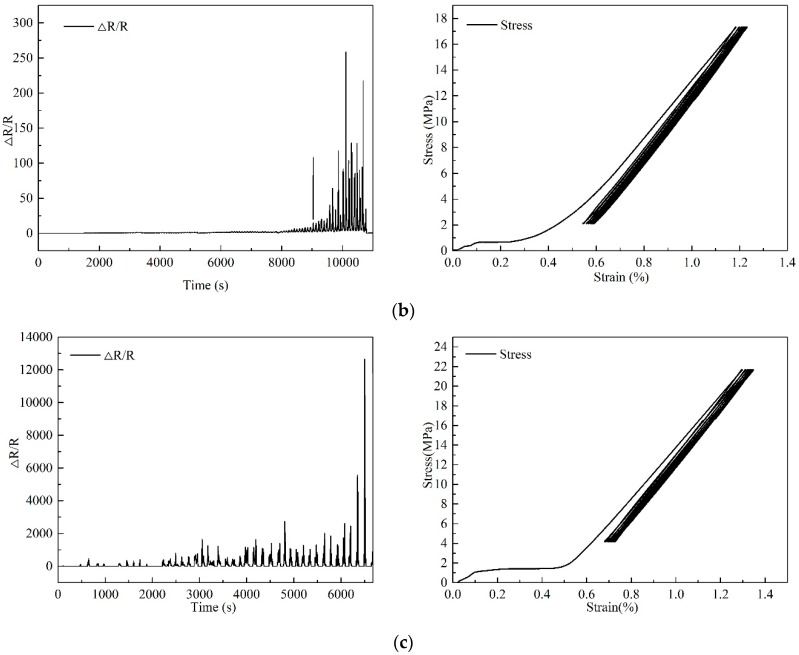
Test results of epoxy coupons subjected to cyclic loading: (**a**) 1.2 wt %; (**b**) 1.6 wt %; (**c**) 2.0 wt %.

In the uniaxial tensile test, nonlinear deformation occurred after the 1/3 of the peak tensile load and the relative resistance-strain curves exhibited a nonlinear manner. In order to investigate whether the relative resistance response could reflect the internal damage of the modified epoxy resins, cyclic uniaxial loading tests were conducted. The maximum tensile load during the tests was about 1100 N and the cyclic loading range was taken between 100 N and the 2/3 of the mean value of the tensile failure load. Due to the poor conductivity of the modified resin when the mixing amounts were 0.4 wt % and 0.8 wt % and the poor dispersion of CNTs when the mix amount exceeded 2.0%, only the mixing amounts of CNTs of 1.2 wt %, 1.6 wt % and 2.0 wt % were chosen for the cyclic loading tests. The tests results are shown in Figure 10.

The stress-strain curves demonstrate that all the specimens experienced the accumulation of plastic deformation during the cyclic loading. The relative resistance response recovered well under each unloading cycle while the peak value increased with the time (i.e., the number of the loading cycles). Non-linearity was obtained at large strains, leading to the modification of the piezoresistive properties under the same loading cycles. This phenomenon, which is regarded as being caused by losing overlapping contact with each other so that tunneling resistance becomes a dominant phenomenon, is similar to the results obtained by [13,14,15]. With the accumulation of plastic deformation, the relative location of the CNTs in the modified epoxy resin exhibited some irreversible changes, leading to the occurrence of damage in the conductive network. The variation of the relative resistance was in several magnitudes and the resistance response became more obvious with the increase of loading cycles. However, the stress-strain curves indicated no abnormal information during the whole cyclic loading. To sum up, the resistance response curve could play a good role on warning the damage of the CNT-modified epoxy resins.

## 4. Conclusions

In this study, CNTs-EP was successfully prepared with good dispersion through the combined methods of three roller rolling, ultrasonic processing and adding surfactant. Then the modified epoxy resins were used to make tensile specimens for force sensitivity tests. Static tests of the resistivity of unloaded modified epoxy resins were conducted. It was found that the mixing amount of 1.2 wt % was the conductive percolation threshold of the modified material, which provided a reference for the following loading tests.

Monotonic and cyclic uniaxial tensile loading tests were conducted respectively on epoxy resin specimens with various mixing amounts of CNTs. It was found that when the mixing amount was lower than the conductive percolation threshold, the junction resistance *R_j_* between adjacent CNTs played a leading role, while when the mixing amount exceeded the conductive percolation threshold, the connected internal conductive network contributed significantly to the conductivity of the modified epoxy resins and segment resistance *R_c_* became more important instead 

For the cyclic uniaxial tensile loading tests, with the accumulation of plastic deformation, the relative locations of CNTs in the modified epoxy resins exhibited irreversible changes, leading to the damage of the conductive network and the dramatic changes (i.e., with several magnitude) of the resistance during the cyclic loading, demonstrating that the resistance response could play a good warning role on the damage of CNTs-modified material. CNTs-EP, with good self-sensing and self-adjusting properties, could act as the main matrix material of fiber reinforced polymer (FRP) reinforcement for use in structures to facilitate damage detection, reinforcement and repair.

## Figures and Tables

**Table 1:** Descriptions of the test materials.

Material	Product Name	Supplier	Material Descriptions
epoxy resin matrix	Forrisio-CFA	Forrisio International Group	Weight ratio of Epoxy resin (A adhesive): curing agent (B adhesive) = 4:1; Bending Strength: 30 N/mm^2^ (+23 °C, 7 days); Density: (1.31 ± 0.1) kg/L; Curing Time: 3~7 days
CNTs	MWCNTs: TNIM8	Chengdu Institute of Organic Chemistry, Chinese Academy of Sciences	Purity >90%; Outer Diameter >50 nm; Inner Diameter: 5–15 nm; Length: 10–20 μm; Special Surface Area >60 m^2^/g; Electric Conductivity >100 s/cm
dispersant	TNEDIS Polymer	Chengdu Institute of Organic Chemistry, Chinese Academy of Sciences	The mixture of butyl acetate and glycol butyl ether, 1.2 times of the mass of CNTs
short carbon fiber powder	UT70-20 carbon fiber	Japanese Toray industries	Used to optimize the conductivity of the modified matrix, the mixing amount of 0.2 wt % and the length of 1 mm

**Table 2:** The gauge factors with various mixing amounts of CNTs.

**Mixing Amount (wt %)**	0.4	0.8	1.2	1.6	2.0
**Gauge Factors**	86.25	122.27	0.23	0.26	1.77
